# Sex as a Biological Variable in Preclinical Modeling of Blast-Related Traumatic Brain Injury

**DOI:** 10.3389/fneur.2020.541050

**Published:** 2020-09-30

**Authors:** Joseph T. McCabe, Laura B. Tucker

**Affiliations:** ^1^Pre-clinical Studies Core, Center for Neuroscience and Regenerative Medicine, Bethesda, IL, United States; ^2^Department of Anatomy, Physiology & Genetics, F.E. Hébert School of Medicine, Uniformed Services University of the Health Sciences, Bethesda, MD, United States

**Keywords:** blast, animal models, sex differences, brain injury, standardization, common data elements

## Abstract

Approaches to furthering our understanding of the bioeffects, behavioral changes, and treatment options following exposure to blast are a worldwide priority. Of particular need is a more concerted effort to employ animal models to determine possible sex differences, which have been reported in the clinical literature. In this review, clinical and preclinical reports concerning blast injury effects are summarized in relation to sex as a biological variable (SABV). The review outlines approaches that explore the pertinent role of sex chromosomes and gonadal steroids for delineating sex as a biological independent variable. Next, underlying biological factors that need exploration for blast effects in light of SABV are outlined, including pituitary, autonomic, vascular, and inflammation factors that all have evidence as having important SABV relevance. A major second consideration for the study of SABV and preclinical blast effects is the notable lack of consistent model design—a wide range of devices have been employed with questionable relevance to real-life scenarios—as well as poor standardization for reporting of blast parameters. Hence, the review also provides current views regarding optimal design of shock tubes for approaching the problem of primary blast effects and sex differences and outlines a plan for the regularization of reporting. Standardization and clear description of blast parameters will provide greater comparability across models, as well as unify consensus for important sex difference bioeffects.

## Introduction

Traumatic brain injury (TBI) is a significant military health problem, with the Defense and Veterans Brain Injury Center reporting ~384,000 worldwide cases from the years 2000 to 2018 in the US forces (1), supporting many studies suggesting a TBI incidence rate of ~20% in service members in Operation Iraqi Freedom (OIF) and Operation Enduring Freedom (OEF) (2, 3). The incidence of blast-related TBI, specifically, rose in military personnel during OIF/OEF compared to previous conflicts due to the increased use of explosive materials [e.g., improvised explosive devices (IEDs), rocket-propelled grenades], and explosions have been determined to be the leading causal agent of TBI in Iraq and/or Afghanistan (4–7). Blast-related injuries have also increased in civilian populations worldwide; conservative estimates from the RAND® Memorial Institute for the Prevention of Terrorism state a fourfold increase in the number of terrorist incidents employing explosive devices between 1999 and 2006, with the number of injuries resulting from those acts increasing eightfold (8, 9).

The increasing participation of women in the US military and the lifting of the Combat Exclusion Policy in 2013, officially giving women eligibility to participate in full combat operations, have placed women at greater risk of sustaining a military-related TBI, including blast-related TBI. The number of women using the Veterans Administration in the United States increased by 46% between 2005 and 2015 (10), confirming female veterans are a rapidly growing patient population. Furthermore, many countries enforce mandatory conscription for women (e.g., Israel, Norway), with women occupying combat positions globally, making TBI in female military populations an international health concern. Although there have been a substantial number of studies comparing outcomes following TBI between men and women in clinical populations, particularly in sports-related contexts [for recent reviews, see (11–13)], specific attention to the potentially special needs of women who have sustained TBI in the military is a growing concern (14, 15). There is substantial evidence that women may be more at risk than men for many neurological and psychiatric conditions following military-acquired TBI [e.g., (16–20)], and it has been recently shown that up to 50% of older female veterans (>55 years old) with a diagnosed TBI also had a psychiatric diagnosis of depression or posttraumatic stress disorder (PTSD) (21). Furthermore, the authors reported that a diagnosed TBI increased the risk of dementia by 50%, and the risk increased twofold for women who suffered from any two of those conditions (21).

Animal models of TBI, including models of blast injuries, have aided in our understanding of the pathophysiology and symptomology of brain injuries for decades [for reviews, see (22–25)]. Since the National Institutes of Health (NIH) mandate requiring inclusion of both sexes in biomedical research (26), the number of preclinical TBI studies including females has increased. There have been several recent reviews on sex differences following TBI in animal models (13, 27–29). However, the majority of translational TBI work employing both male and female animals has been performed with more severe and/or surgically invasive TBI models such as controlled cortical impact [CCI; e.g., (30–35)], fluid percussion injury [FPI; e.g., (36, 37)], or repetitive concussive brain injury [CBI; e.g., (38, 39)]. Although blast injury models have been studied in male animals of many species (23, 40), there is a near absence of female inclusion in blast models of neurotrauma.

The purpose of this review is to discuss TBI, particularly as inflicted by a blast event, in the context of sex as a biological variable (SABV). What are known about the functional consequences of military-acquired blast TBI are discussed, followed by a description of the present experimental approaches that have had utility in manipulating sex chromosomes and gonadal steroids as independent variables. Dependent variables, including pituitary, autonomic, vascular, and inflammation factors, are then discussed because these focus on the most reported systems that are perturbed by blast. Finally, relevant to the study of blast and SABV in preclinical studies, the review makes an appeal for investigators to apply the highest-quality experimental principles, because the study of SABV in this field is complex and requires the derivation of the uppermost-quality information for asking further questions and laying the groundwork for translational relevance. Suggestions and guidelines are provided for the use and reporting of sufficient information about blast animal models that will aid in the interpretation of data and generation of conclusions.

## Consequences of MTBI In Military Populations

### Mild TBI Symptoms

Mild TBI (mTBI) as a result of an explosion often leads to symptoms that are well-studied in military populations. The symptoms are most often short-term, resolving within 7–10 days, and often include physical (e.g., headache, dizziness, nausea), cognitive (e.g., memory and concentration problems), and behavioral (e.g., anxiety, irritability) complaints (41, 42). However, a small percentage of patients (~10–25%) will have symptoms persisting >3 months and will be diagnosed with postconcussive syndrome (PCS), which can include a variety of symptoms ranging from psychiatric (anxiety and depression) to physical (headache, fatigue, dizziness) and sleep disturbances, among others (43, 44).

In addition to potential long-term symptoms following mTBI, there is a clear link between military-acquired TBI and PTSD [e.g., (4, 45, 46)]. PTSD has many overlapping symptoms with mTBI and PCS (e.g., irritability, fatigue, poor sleep, memory, and attention problems), but PTSD is often referred to as an “abnormally sustained stress response” with added symptoms of nightmares, hyperarousal, avoidance, and re-experiencing phenomena (47). Fully understanding the relationship between TBI and PTSD, and whether blast-related TBI carries a higher risk of a PTSD development than TBI incurred by other mechanisms, has been a challenge for researchers, and discussion of this complex topic is beyond the scope of this article (47). However, blast exposure clearly puts individuals at risk of the development of PTSD (4, 48–52) and other psychiatric conditions such as depression (4, 48–50).

### Comparisons of Blast vs. Non-blast TBI

It has been noted that the study of head trauma in military populations is difficult. Although blast has been the most common cause of mTBI in recent conflicts, military personnel are simultaneously at risk of TBI from other causes such as motor vehicle accidents, rigorous training exercise and sports, falls, fights, etc. (53), making it possible or even likely that a subject in a study has sustained more than one TBI of different types (blast, concussion, etc.) over their deployment, or earlier in their lifetime. Greer et al. (54) recently conducted a meta-analysis of the literature comparing clinical and functional outcomes in blast and non-blast TBI in US OIF/OEF service members and veterans. For most outcome measures studied (i.e., vision loss, vestibular dysfunction, depression, sleep disorders, alcohol abuse), there were no differences between blast and non-blast TBI groups. For other outcome measures (i.e., PTSD diagnosis and symptom severity, headache, hearing loss, and neurocognitive function), results were inconsistent (54). Thus, there were no functional measures that could be definitively linked to blast-induced TBI. Importantly, the authors also reported that the majority of the studies used varying definitions of “blast” and “non-blast” injury, and there was often little information about the blast injury, including how close the individual was to the explosion, if he/she was in a vehicle or dismounted, whether there was a loss of consciousness, additional trauma, etc. (54). Indeed, it has been noted that the majority of sustained blast injuries include a mixture of secondary and tertiary injuries (sometimes called “blast-plus” see *Blast Events*), making the contributions of the primary blast wave to the subsequent outcomes difficult to clearly establish (55, 56).

Several publications, however, employed *in vivo* imaging and discovered morphological or functional differences between blast-exposed cases and other forms of TBI. An initial key observation was evidence of white matter tract changes. Davenport et al. (57) examined white matter tract integrity with diffusion tensor imaging (DTI) in service members with a reported blast-related TBI and with no reported blast exposure or signs of mTBI. Twenty subcortical white matter tracts were evaluated for differences in fractional anisotropy (FA; a measure of white matter integrity). Ten white matter tracts had significantly lower FA measures in the cases with blast-related TBI. The authors noted that the analysis required a close assessment of the regions of interest, and the differences were diffuse and widespread, and the specific tracts with alterations varied across the cases. Levin et al. (58) performed DTI assessments and an extensive characterization of veterans and service members with exposure to blast and a group with no TBI or blast-exposure history. No differences were found between the groups, but FA measures in some brain regions were associated with impairments of verbal memory. DTI was employed by MacDonald et al. (59) to compare alterations in service members who had a history of blast exposure, as well an additional blast-related trauma (e.g., impact with objects, a fall, or motor vehicle crash). The control group in this study comprised individuals with blast exposure and other injuries, but who had not received a TBI diagnosis (59). DTI changes were seen in the service members with a diagnosis of TBI. Alterations in DTI were employed by Bazarian et al. (60) to assess the relationship of white matter tract alterations and mTBI with the severity of PTSD symptoms. FA measures were associated with blast exposure, and PTSD severity was associated with stress symptoms and abnormal DTI, but not with an assessment of mTBI, suggesting DTI changes were observed in “subclinical TBI” cases (60). Taber et al. (61) compared white matter changes in veterans with primary blast exposure (but no TBI symptoms), individuals with reported primary blast exposure consistent with no TBI symptoms or a lack of signs to indicate mTBI, or no exposure to blast. Compared to veterans who had no history of blast exposure, veterans who had sustained a blast event, with or without a diagnosis of TBI, were found to have lower FA and higher radial diffusivity [a general correlate of myelin damage; (62)]. Similar to the observations of Davenport et al. (57), the changes were heterogeneous and widely dispersed. A significant observation from this report is that the changes were seen even in participants with no presenting TBI complaints. Trotter et al. (63) also examined white matter integrity in veterans with a history of blast exposure or with no reported incidence. The participants were 19–62 years of age and had sustained severe blast injury and were compared with service members who had no exposure to blast. In each group, some of the participants had a diagnosis of TBI (69 and 53%, respectively). Alterations in white matter integrity were associated with the intensity of blast exposure, and decreased degree of FA was associated with the number of years since the most severe blast injury.

Several studies have employed functional magnetic resonance imaging (fMRI) to assess cerebral blood flow as a correlate of cerebral activity. Han et al. (64) found blast-related TBI disrupted resting-state cortical network function compared to participants who also had experienced blast exposure but were not diagnosed with TBI. Robinson et al. (65) described a difference between functional connectivity within components of the default mode network when service members were close to a blast (<10 m) compared to individuals located at a site that was farther from the blast. fMRI was used by Fischer et al. (66) while participants attended to the Stop Signal Task, a measure of response inhibition/impulse control (67, 68). Participants included individuals who sustained blast-related TBI, control (uninjured) military personnel, civilians with no TBI, and civilians with non-blast TBI. fMRI activation was lower in service personnel during correct inhibition responses compared to military controls personnel in brain regions associated with response inhibition and the default mode network. Interestingly, the service members with blast-related TBI exhibited greater activation than controls in trials where the respondent failed to appropriately inhibit their response during the Stop Signal Task. In contrast, non-blast civilians displayed an opposite process where TBI civilians had less activation compared to civilian controls. As noted later (see *Vascular Alterations From TBI*), vascular changes are noted in clinical and preclinical studies of blast effects. Sullivan et al. (69) applied arterial spin labeling to assess possible changes after blast. An increase in the total number of blast exposures was associated with increased cerebral perfusion, but there was no noted relationship to blast proximity or a diagnosis of mTBI or PTSD (69).

Several trends, then, are gained from MRI. First, alterations in white matter integrity have been observed, and the findings suggest these cases exhibit diffuse changes and variability in the location of changes (60, 63, 65, 66), although there is perhaps overlap with impact-related mTBI cases [e.g., (61, 70, 71)]. Second, there are intriguing hints of differential metabolic, gray matter, alterations, particularly for fMRI analyses where the injuries from blast are associated with milder impairments on some performance tests. The finding of a variance in the default mode network response pattern in blast and non-blast TBI cases may point the way to differences in mechanism (66). Lastly, some observations suggest MRI differences are observed in cases where clinical diagnoses of mTBI are not reported (60, 61). Relative to sex differences (most of the cited studies had few or no female participants), white matter alterations and fMRI changes may provide important clues, including potential differences related to activity during performance tasks (66).

### Women and Military-Related TBI

Research on military health and blast-induced brain injuries has largely focused on males, as they have historically made up the majority of service members of the US military and have been more likely to occupy combat roles. However, ~300,000 female service members were deployed to Iraq and/or Afghanistan between 2001 and 2013 (72), and they currently make up 15% of active-duty armed forces. The incidence rate of TBI in deployed women is estimated at ~10%, about half that of deployed men (16, 20, 45, 73–75). It should be noted that the causes of TBI in military women differ substantially from those of men; intimate partner violence (including physical and sexual assault) is recognized as a significant risk factor for TBI in military women compared to their nonmilitary peers (14, 19, 76). However, the increasing participation of women in combat operations during deployment in recent years continues to put them at greater risk of combat-related TBI (77), and like male service members, blast events have been identified as the greatest cause of combat-related injuries, including mTBI, in women during OIF/OEF (16, 78).

Overall, following combat-related mTBI, women are likely to suffer the same symptom clusters as their male military peers, such as PCS, PTSD, psychiatric complaints (i.e., anxiety and depression), and somatic symptoms (e.g., vestibular and somatosensory dysfunction) (16–19, 45). However, several studies comparing the outcomes of male and female service members following mTBI have reported differences in the frequencies of specific diagnoses and symptoms between men and women. In a recent scoping study describing the literature addressing gender differences in outcomes following TBI in military populations, Cogan et al. (19) identified 29 relevant articles from 2000 to 2018. One clear conclusion was that women are very underrepresented; most of the studies were not specifically focused on gender differences, and women represented <20% of the sample. The most consistent finding to date was that following a TBI, females in the military are subsequently more susceptible to depression than male service members and veterans (16, 19, 45, 79, 80).

In addition to depression, there is evidence that female service members may have increased susceptibility to anxiety disorders and/or PTSD following mTBI. The literature describing gender differences in PTSD symptoms in military personnel is relatively broad and reports mixed results, possibly as a result of variations in the definition of TBI or methodological differences (18). In an earlier study, Iverson et al. (16) reported that although men were more likely to be diagnosed with PTSD alone following mTBI, women were more likely to have PTSD with comorbid depression. Women were also more likely to suffer from a non-PTSD anxiety disorder and/or to have more than one psychiatric diagnosis compared to men. By subsequently adjusting the model for blast exposure, the authors were able to provide some insights into the potential specific contributions of blast injury to sex differences in outcomes following TBI; there were no longer differences in the likelihood of a PTSD diagnosis alone, frequency of non-PTSD anxiety disorders, or diagnosis of more than one psychiatric condition (16). These results suggest that blast may uniquely contribute to the female susceptibility to anxiety disorders and PTSD with comorbid depression and to the diagnosis of multiple psychiatric diagnoses.

Because of the complexity of ascertaining relevant variables for blast mTBI etiologies, it is important to supplement the clinical literature by applying preclinical animal research. To further understand the role of sex-related variables as proximate causes for sex differences, animal modeling enables greater control of conditions and the ability to more invasively explore cellular response mechanisms from blast exposure. Following an overview of approaches to the study of SABV as an independent variable for blast TBI preclinical work, there is a summary of what is presently known concerning blast bioeffects on pituitary and the hypothalamic–pituitary–adrenal (HPA) axis, the autonomic nervous system, the vasculature, and inflammation.

In addition to the NIH mandate regarding consideration of SABV in clinical and preclinical research, a second policy relates to scientific rigor by employment of preclinical experimental practices that derive valid and reliable findings to adequately address research gaps, set the stage for discovering important mechanisms underlying sex difference and properly modeling translational testing (81–83). Accordingly, there is discussion for a second important feature of preclinical blast research related to principles for application of shock tubes, the most common approach for preclinical modeling.

## SABV in Blast MTBI Research

### SABV

Approaches to the study of SABV in animals has been clearly articulated in several reviews (84–87). With respect to TBI, data summarized by Gupta from 43 studies that examined sex differences, using many different outcome criteria following a variety of TBI models (CCI, FPI, CBI), concluded that females fared better in 55% of the studies, and none indicated males had a better outcome (13). Their Table 2 included a single preclinical blast paper by Russell and colleagues; reviewed below in *HPA Axis Dysfunction in Laboratory Animals After Blast*. The review by Rubin and Lipton (29) of 50 articles found high variability in outcomes, but they too concluded that generally females fared better after injury by FPI, CCI, and weight drop.

For preclinical study of SABV and blast effects, Table 1 summarizes the main dimensions for investigation of sex as an independent variable. The aforementioned publications regarding experimental design are excellent summaries, and the most salient issues related to sex chromosomes and steroid hormone status are discussed. Subsequently, what are perhaps the most relevant bioeffects of blast exposure, as dependent variables, are outlined, with particular attention to previous preclinical findings in blast TBI experiments.

**Table 1:** Approaches to sex as a biological variable in preclinical blast research.

**Sex-related variable**	**Experimental approach**	**Relevancy**
Sex chromosomes	Male testis-determining gene, Sry mouse model, X^*^ mouse strains	Permit study of the impact of *Sry* and possibly other Y chromosome–encoded genes; X chromosome (single X, XX, models) permit study of the genetic load of the X chromosome
Estrous cycle factors	Assessment and comparison of endocrine status	Ascertainment of ovarian cycle effects with injuries sustained at a particular stage of the ovarian cycle may lead to insights regarding differential effects on outcome
Gonadal hormone status	“Endocrine ablation” by gonadectomy; hormone replacement	Assess gonadal steroid effects upon dependent variables. Other factors include reproductive status, possible relevancy to contraceptives, hormone replacement therapies, steroid or anabolic steroid use, exposure to endocrine disrupting chemicals (e.g., phthalates, bisphenol), menopause, eating disorders, intense physical activities

#### Gonadal Hormone Effects

Some evidence suggests there is no relationship for estrous phase as a significant impact on outcomes after TBI (88–90). However, potentially subtle endocrine factors that have important mechanistic ramifications may be overlooked when studies do not account for potential differences related to estrous cycle stage in females (84). There is strong evidence supporting the neuroprotective roles of estrogen and progesterone, suggesting that female animals may be more resistant to the deleterious effects of injury during the proestrous phase of the cycle, when levels of hormones are at their highest. If there is specific interest in estrous cycle effects, initial studies to evaluate SABV related to blast can be directed to the basic hormone status of laboratory animals by assessment of menstrual cycle. Becker et al. (84) suggest the experimental design could compare male rodents with four groups of females, one group at each stage of the estrous cycle. This allows the researcher to determine if sex and/or the variable levels of steroid hormones across the estrous cycle affect the dependent variable(s) in question. To evaluate the estrous cycle stage, vaginal smear examination should be performed daily, and it has been suggested to perform the examination for at least 8 days immediately prior to an experiment (91). Likewise, for better assignment to hormone status, it is suggested that animals be excluded should they not exhibit regular cyclicity (91). When experimental questions relate to the estrous cycle, these are important considerations, and care should be taken in defining estrous cycle stage, as hormone levels change very rapidly during the day, particularly during proestrous when progesterone levels are peaking (84).

There has been speculation concerning the significance of estrous status in laboratory animals, and some have warned that this is challenging in rodents with shorter cycles where there is inherent variability, even across time of day. Disregarding cycle effects was considered problematic because females may exhibit greater data variability, perhaps complicating interpretation. Alternatively, it is argued that employing female animals at random/cycling stages of estrous more accurately represents the clinical condition. Nonetheless, comparisons of measures in female and male mice and rats suggest variability may not be a significant factor (92–94). Shansky (94) has pointed out other related factors that affect hormone status should be considered, including housing conditions, which was found to affect variability and that group housing of male rodents can alter testosterone levels. Circadian or seasonal factors may also come into play as a variable (95, 96). In addition, some reports relate changes due to female hormonal status, and findings from an initial study of sex differences may suggest the need for closer examination of estrous cycle as an important variable.

#### Sex Chromosomes

The pioneering observations of Nettie Maria Stevens documented the spermatozoa of *Tenebrio molitor* mealworms contain nine similarly sized chromosomes and a smaller chromatin element related to male offspring; in contrast to spermatozoa with 10 chromosomes of equal size associated with female progeny (97). Thus began the intriguing pursuit of sex chromatin differences, subsequent XY nomenclature, and attention to their potential significance in sex-linked disorders (98). The genetic sex of neurons, glia, the cerebral vasculature, and other support cells of the central nervous system and the response of peripheral organ systems to blast injuries are important variables for investigation. Potential differences attributable to sex chromosome effects relate to X chromosome exclusion, where in female progeny the maternal X chromosome (X_M_) or the paternal X chromosome (X_P_) is silenced by X chromosome inactivation (XCI) to partially rebalance the level of expression (99). XCI leads to a mosaic expression pattern in females where the cells in an organ express X_M_ or X_P_, although across the female population there is further complexity related to the degree of mosaicism and that a proportion of genes on the “silenced” X chromosome escape inactivation (99). Genes encoded on the male Y chromosome may also have differential effects on cell phenotype and responses to injuries if the pathways are not also homologously encoded on X chromosomes (84).

The mammalian Y chromosome encodes the testis-determining gene, *Sry*, which initiates testes formation and spermatogenesis, as well as a small number of additional genes with X-linked homologs that, in females, escape XCI (99). One approach to understand the differential contributions of hormone effects and sex chromosome effects employs the “four core genotype” design in mice (100). The four genome design includes deletion of *Sry* from the male Y chromosome and insertion of the gene in an autosome. This allows the creation of four genomes: (1) an XY complement with the *Sry* gene for XY mice with testes; (2) an XY complement without *Sry*, resulting in XY mice with ovaries; (3) an *Sry* mouse with the gene incorporated into an autosome resulting in an XX mouse with testes; and (4) an XX mouse with no copy of *Sry*, resulting in XX mice with ovaries. The mice with similar gonadal forms then permit investigation of the sex chromosome complementation (XX vs. XY) in the context of gonad-related hormonal status (101). To date, this paradigm has not been employed in preclinical blast studies. However, sex chromosome differences have been associated with pathological effects. Li et al. (102) used a cardiac ischemia/reperfusion model and found infarct size was greater with two X chromosomes, independent of gonadal status, compared to XY mice. A second study in this report employed the XY^*^ mouse model that allows comparisons for the number of X chromosomes and likewise found XX mice exhibited poorer recovery than 1X females (102). Other sex chromosome–related models available, and more complex genomic analyses can be applied (98, 103, 104). No preclinical studies have examined sex chromosome effects after blast injuries.

#### Gonadal Steroid Effects and Steroid Receptors

Despite some debate regarding menstrual cycle status as a significant factor in TBI outcome (see *Gonadal Hormone Effects*), gonadal steroid action has been a main, classic focus for the study of sex differences. This is particularly relevant because there is strong evidence of neuroprotective roles of estrogen and progesterone after a range of brain injuries (105–108). As an initial procedure for the study of SABV, Becker et al. (84) describe a standard “two-step approach” for the study of SABV that comprises an initial effort to determine steroid action by gonadectomy, followed by procedures to provide replacement of the hormone. In the first procedure, male and female animals receive a gonadectomy as a comparison to endocrinologically intact animals. Separate groups of animals receive a sham procedure where the identical surgery is performed to externalize the gonads followed by replacement *in situ*. At specified times after the procedure, animals are utilized in the study. If the gonadectomy resulted in an experimental change for the variables under study, the second step is undertaken where gonadal steroids are administered to gonadectomized animals, whereas a control group receives similar treatment by administration of the vehicle diluent for the hormone(s). Becker et al. (84) note that a third group can be incorporated in this step by including gonadally intact animals as a comparison. Differences are preliminarily interpreted as indication of a gonadal steroid contribution to the biological process under study.

There are additional variables to consider for the two-step approach, including the timing of testing (e.g., surgical treatment or hormone replacement following brain injury) following gonadectomy in the first step as well as after hormone replacement in the second phase of the study. Experienced investigators suggest that the administration of gonadal steroids should be monitored to ensure the replacement procedure provides levels of steroid within the physiological range. Further studies can employ the same approach with compounds that block steroid synthesis or that disrupt steroid receptor effects, or to determine the role of clinically relevant intervening effects on steroid action such as contraceptives. Maintenance on the contraceptives, desogestrel and drospirenone, e.g., was found to reduce the severity of stroke neuropathology in ovariectomized mice (109). Finally, further experiments can be performed to determine the role of specific steroid receptors subtypes. In one of the first articles to explore the role of the two estrogen receptors (ERs), Dubal et al. (110) employed a stroke model that occluded the anterior cerebral artery in ovariectomized mice. Some of the mice were given estrogen replacement in Silastic capsules or the vehicle alone, sesame oil. In the ERα knockout mice provided with physiological levels of 17β-estradiol, level of injury was equivalent to what was observed in wild-type mice or ERβ knockout mice that were not provided with estradiol, indicating the α receptor mediates the neuroprotective effects of estrogen.

### Bioeffects of Blast Exposure

A second level of inquiry relates to blast-related mechanisms—the dependent variables—that may be differentially affected by sex differences. Outlined below are the most salient biological effects known to date for the impact of blast exposure relevant to SABV. The discussion for some effects begins with clinical descriptions, but some of the reportage concerns findings with non–blast-related methods that may help point to relevant effects, including sex-relevant differences of pituitary and HPA axis function, and blast effects on the autonomic nervous system function, the vasculature, and inflammation. Although researchers often focus on a single dimension of outcome, investigators have recognized that TBI consequences demonstrate it manifests as a systemic condition (111, 112). Likewise, it is probably a significant truism that blast exposures should be considered a polytrauma. High-energy shock wave exposure injures, or at least perturbs, all organ systems, leading to complex, reciprocal interaction between peripheral organs and tissues and central nervous system networks.

#### Clinical HPA Axis Dysfunction After TBI and Blast Exposure

Although more studies of military-acquired, particularly blast-related, mTBI in women are required, a picture is emerging of a gender dichotomy in the stress response following mTBI. There are clear sex differences in non-TBI civilian populations in the lifetime susceptibility to depression and anxiety disorders (113, 114), as well as evidence from the civilian literature that women may be more susceptible to psychiatric disorders following mTBI [e.g., (115–118)], although data are not entirely consistent (13). Anxiety and depression, as well as PTSD, are linked to the HPA axis, the major neuroendocrine system that controls responses to stress (119–122).

The primary stress hormone is cortisol (CORT; corticosterone in laboratory rodents), which is released by the HPA axis when activated by a physical or psychological stressor. The stress response is characterized by release of corticotropin-releasing factor (CRF) from the paraventricular nucleus (PVN) of the hypothalamus, which binds to CRF receptors on the anterior pituitary gland. The anterior pituitary gland secretes adrenocorticotropic hormone (ACTH) into the bloodstream, where it reaches the adrenal cortex and binds to receptors to stimulate the synthesis and release of the steroid hormones, glucocorticoids (e.g., CORT), and mineralocorticoids (e.g., aldosterone). Steroid hormone receptors are located throughout the brain [see (123) for more detailed review], including limbic regions involved in emotion and responses to stressful stimuli.

Clinical studies have demonstrated HPA axis dysfunction in a proportion of individuals several months to years following mild to moderate TBI (119, 120, 124). The pituitary gland is especially vulnerable to damage, with multiple potential syndromes resulting from hormonal deficiencies (e.g., hypogonadism, hypothyroidism, central diabetes insipidus) (125, 126). Little is known about HPA axis disruption following blast injury, although there are reports indicating decreases in pituitary function up to 2 years following blast-related mTBI (127) or moderate to severe blast TBI (128). A follow-up study found that pituitary dysfunction following blast-related mTBI was associated with increased neuropsychiatric symptoms (i.e., anxiety, irritability) compared to individuals with mTBI and normal pituitary hormone levels (129).

#### HPA Axis Dysfunction in Laboratory Animals After Blast

In addition to the insight provided by clinical studies, translational studies employing animal models have allowed further probing of the pathological underpinnings of TBI-induced HPA axis dysfunction (122, 130–136), as well as the use of validated and controlled behavioral paradigms for measuring anxiety- and depressive-like symptoms following experimental TBI (137, 138). Serum levels of ACTH have been shown to decrease 1 month following blast injury in male rats, followed by an increase at 3 months postinjury, suggesting a biphasic blast-induced hypothalamic–pituitary dysfunction (139). Recently, Zuckerman et al. (140) evaluated the CORT response in male rats at more acute time points following blast exposure. Animals exposed to blast had elevated CORT levels 3 h following blast that returned to baseline within 5 h. However, rats with a PTSD-like phenotype, as assessed by their behavior 1 week following injury in the elevated plus maze (EPM; a test for anxiety) and the acoustic startle response (tests for heightened responses to a sensory stimulus), had blunted CORT responses compared to blast-exposed rats with a “well-adjusted” phenotype (140).

Although investigators have recently turned their attention to sex factors in a variety of TBI models [for reviews, see (13, 27–29)], preclinical studies of blast effects, and specifically on the effects of blast on HPA axis function and/or the development of anxiety and depressive disorders, remain essentially nonexistent. In fact, Russell et al. (136, 141) are the only investigators to date to assess sex differences in the effects of blast-induced TBI in an animal model. In two publications, they reported the effects of mild blast TBI on central and HPA axis function (136) and on CRF receptor gene expression and anxiety-like behaviors (141). Sex differences following exposure to blast overpressure in the advanced blast simulator (ABS; described in more detail below) were reported in both studies.

First, the authors employed a restraint-induced stress model and demonstrated that while blast injury increased the restraint-induced rise in CORT levels in males, the opposite effect was observed in female mice, with blast attenuating CORT levels in restrained animals compared to sham-treated mice. Blast did not alter CORT suppression in the dexamethasone-suppression test or affect the expression of pituitary or adrenal genes involved in ACTH or CORT synthesis or secretion, suggesting a central disruption in feedback, rather than a peripheral effect, as the more likely source of the sexually dimorphic response to injury. Examining potential central nervous system sources, it was first determined that there were no effects of blast injury in either males or females on mRNA expression of mineralocorticoid and glucocorticoid receptors at central feedback regulation sites: the PVN or other brain limbic structures [e.g., amygdala, hippocampus, bed nucleus of the stria terminalis (BNST)]. However, a restraint-induced increase in CRF neuron activation was differentially altered by blast injury in male and female mice: in males with restraint treatment, blast (compared to sham treatment) reduced CRF neuron activation in the PVN; in females, restraint-treated mice receiving blast treatment had increased levels of CRF neuron activation in the PVN. Retrograde tracing determined that there was a TBI-related decrease of CRF neurons in female mice primarily in preautonomic (non-neuroendocrine) neurons in the PVN, suggesting a decreased use of the preautonomic system in dealing with stressors, leading to a possible blast-induced disruption in CRF outputs to brainstem structures regulating autonomic function. There were no blast-induced changes in the percentage of activated CRF neurons that were endocrine projecting or preautonomic projecting in male mice, and the authors hypothesized that disruption in limbic structures of the HPA axis may result from blast-induced TBI.

A second study was designed to measure changes in the expression of CRF receptor subtypes 1 and 2 (CRFR1, CRFR2, respectively) in limbic structures following blast-induced brain injury in male and female mice, as well as to assess the sex-dependent effects of blast on anxiety-like behaviors (141). CRFR1 is widely distributed throughout the brain, and blocking these receptors reduces psychiatric symptoms, whereas expression of CRFR2 is more localized, and activation of these receptors dampens stress responses (142, 143). Blast did not affect CRFR1 expression in either male or female mice, but the injury altered CRFR2 expression in limbic structures in a sexually dimorphic way. The restraint-induced increase in CRFR2 expression was reduced by blast injury in the dorsal hippocampus in females, and in the amygdala and anterior BNST of male mice. In addition, in males, blast injury increased basal CRFR2 (non–restraint-induced) expression in the ventral hippocampus. These changes in CRFR2 expression were paralleled by decreased time spent in the open arms of the elevated plus maze by both males and females, indicating elevated levels of anxiety. The authors suggest that the increase in anxiety following blast injury results from the downregulation of CRFR2 and reduced compensation for the angiogenic effects of the CRFR1 (141). This hypothesis is supported by the observation that CRFR2 knockout mice have increased anxiety-like behaviors (144). Furthermore, the sex differences observed in regional changes in CRFR2 expression post-TBI suggest that male and female mice employ different limbic circuits to cope with the effects of TBI.

#### Autonomic Nervous System Function After TBI

During the acute period following TBI, systemic effects appear to result from excessive catecholamine release and subsequent autonomic dysfunction. In more severe cares, autonomic dysfunction leads to transient episodes of paroxysmal sympathetic hyperactivity (PSH), which includes tachycardia, hypertension, hyperthermia, spasticity, and tachypnea (145–147). A recent review by Baguley and colleagues (148) provides support for an excitatory:inhibitory ratio model. TBI that includes damage to the mesencephalon results in the loss of descending inhibitory inputs to spinal pathways, resulting in acute, non-nociceptive stimulatory, autonomic overreactivity. Fernandez-Ortega et al. (149) studied 179 severe TBI patients and found ~10% of the sample exhibited PSH; all were male patients. For blast wave exposure, acute autonomic responses are elicited from pulmonary injuries (“blast lung”), an organ particularly susceptible to damage, resulting in cardiorespiratory distress [c.f., (150)]. Blast lung symptoms include bradycardia and prolonged hypotension, as well apnea episodes followed by rapid, shallow breathing, where bradycardia and hypotension are a result of vagal reflex responses, whereas the hypotension results from autonomic changes, direct heart damage, and the acute release of the potent vasodilator, nitric oxide [cf., (151, 152)]. Pulmonary hemorrhage and edema, as well as later proinflammatory mediators, are activated, which further compromise pulmonary function (152, 153). To date, there appear to be no publications that have explored PSH after blast injuries, as well as no studies of PSH and more severe cases of blast lung that compared the sexes.

Evidence for persistent cardiovascular changes after milder cases of TBI has been reported, with alterations in cardiac rhythm variability providing an overall, integrated indicator of autonomic function (154–156). In milder insults, it is hypothesized that injury results in subtle anatomical lesions in central autonomic networks that give rise to functional changes seen in potentially unhealthful or lethal cardiac irregularities (154). Manifestation of dysregulation may only be evident with close physiological monitoring of autonomic response challenges, such as standing, but less conspicuous changes are also reported during the resting, supine state (154). The six studies reviewed by Bishop et al. (156) appear to have focused on male athletes. "However, Hilz et al. (154) reported on three females and 17 males. La Fountaine et al. (157) studied three subjects (two females, one male), and Senthinathan et al. (158) studied seven females and four males, but none of the reports analyzed sex differences. For blast injury, there appear to be no studies that have examined SABV for cardiac variability or other autonomic changes. However, SABV for autonomic differences is important. In general, females exhibit greater vagal activity, whereas males generally manifest higher sympathetic activity [cf., (155) for review], and uninjured females exhibit a greater baseline of heart rate variability (159). Likewise, gonadal hormones are known to modulate autonomic nervous system networks, where, e.g., estrogen administration to male and ovariectomized rats increased cardiac baroreflex response (160, 161).

#### Vascular Alterations From TBI

##### Evidence of physical damage

Perhaps on par with reports of significant changes in neuroinflammation after blast exposure (see below), vascular alterations from blast exposure have received the greatest research attention. A particularly vulnerable organ to blast exposure is the pulmonary system, where more energetic shock waves result in significant lung contusions and accompanying autonomic dysregulation (see *Autonomic Nervous System Function After TBI*) and further trauma with leukocyte recruitment and the release of proinflammatory signals [(153) and see *Inflammatory Factors*]. However, in addition to lung response, other effects are observed throughout the vasculature.

An oft-cited hypothesis for the initial physical effects for brain injury relates to “hydrodynamic pulse through venous vasculature,” a mechanism purported mainly by Cernak (162, 163). Briefly, the energy from a blast exposure is transferred to the body causing a rapid alteration in abdominal, thoracic, and central venous pressure. Cernak (163) cites Gelman's (164) report that ~70% of blood volume in humans is in the venous compartment compared to 18% in arteries and the remaining 3% in terminal arteries and arterioles. The abrupt pressure change in the arterial and venous vasculatures further contributes to rapid pressure changes in the common carotid artery and inferior vena cava, inducing fluid sheer stress that may result in platelet-activating factor–induced neutrophil activation (163, 165), as well as additional complex interactions (163). Some reports have described peripheral organ damage for endothelial barriers (166). However, a blast-mimicking pulse to the thorax of anesthetized rats also causes widespread neuroinflammation, evident by tumor necrosis factor α in perivenular regions in the brain and activated microglia and macrophages adjacent to veins (167). Investigations in rodents also have described cases of blast exposure resulting in signs of minor cerebral injury, including instances of tears of penetrating cortical vessels, microhemorrhages, swelling, and end-feet degeneration of perivascular astrocytes (168–170). All of the aforementioned studies have employed male laboratory rodents. Finally, the injury effects of blast exposure, having an impact on central functions, including central autonomic networks and immunomodulation, are potentially complex interactions where peripheral injuries affect cerebral functions and reciprocal links from brain to peripheral organs (112).

##### Cerebral vasospasm

A common sequelae to blast exposure is cerebral vasospasm (171). The publication by Armonda and colleagues was one of the first clinical reports to describe this phenomenon, most frequently evident in more severe cases (172). Of interest was that the vasospasm occurred as a delayed phenomenon, peaking about 2 weeks after blast injury and lasting for at least a month, the length of the study (172). In the report of Armonda et al., the sex of the casualties was not reported, but ~50% of the injured patients exhibited vasospasm. Although there have been no descriptions of sex differences in vascular reactivity after blast exposure, young females admitted to hospitals after impact-related TBI were found to show vulnerabilities. Czosnyka et al. (173) observed that after accidents young women exhibited greater cerebral hypertension and reactivity. In severely injured patients [requiring intubation, mechanical ventilation, intracranial pressure (ICP) monitoring], Sorrentino et al. (174) likewise reported a vulnerability, where a more favorable outcome was observed if younger female patients had lower ICPs and lower pressure–reactivity index (PRx; a measure of cerebral autoregulation), perhaps in line with reports of higher vulnerability in females (and older patients) with ICP. Hamer et al. (175) recently reported observations in young athletes (19–21 years of age) who had sustained single or multiple concussions. Males were found to exhibit lower cerebral blood flow in temporal regions, whereas female athletes with a history of concussions were not different from uninjured females. However, females who had sustained multiple concussions, compared to women who sustained a single concussion, exhibited lower cerebral blood flow in the left anterior cingulum and right cerebellum and middle occipital gyrus. The role of vasospasm was not addressed, but the authors speculate the alterations may relate to long-term central metabolic activity changes or perhaps a loss of cerebral volume from injuries.

Alterations in vascular reactivity have been reported in preclinical studies, but studies are skewed to males. As noted by Mollayeva et al. (176), there appears to be a discrepancy in the preclinical literature, where more frequently female laboratory animals exhibited better cerebral hemodynamics after TBI. A study by Armstead et al. (177), e.g., studied pial microvascular responses after fluid percussion injury in piglets. Following injury, male piglets were observed to exhibit greater reductions in pial artery diameter, cortical cerebral blood flow, and cerebral perfusion pressure, as well as greater elevation of ICP after injury (177).

#### Inflammatory Factors

In addition to signs of vasculature damage, inflammation is often observed acutely with proximate tissue damage, as well as over the long term as a secondary consequence (27). Cernak et al. (178) pioneered in describing acute systemic inflammation after blast exposure. “Local” effects of blast exposure were observed, including the activation of eicosanoids—bioactive, locally released immune system signals (179). This group sampled plasma from 65 blast-injured male personnel, using an inclusion criteria of signs of lung injury, and found higher blood levels of thromboxane A2, prostacyclin (PGI2), and sulfidopeptide leukotrienes, in comparison with 62 patients who sustained similar levels of injury severity, but had not sustained blast exposure. Subsequent studies using whole-body imaging in mice found elevated myeloperoxidase activity, a measure of activated phagocytes, throughout the gastrointestinal tract, lungs, and brain that persisted for at least 1 month, with central nervous system response suggesting a higher expression at 1 month, the last time point assessed (180). Similar observations have been reported from blast trauma in the lungs and brains of male rats (181). Gorbunov et al. (153) described pulmonary contusions from shock wave exposure (alveolar rupture and blood extravasation) and the release of proinflammatory signals, including macrophage inflammatory protein-2, interleukin 6 (IL-6), monocyte chemoattractant protein-1, and cytokine-induced neutrophil chemoattractant-2 [summarized in Gorbunov et al. (153)].

Central nervous system inflammation is a key variable after TBI. Investigators hypothesize brain injury leads to chronic, lower-level neuroinflammation that results in insidious neurodegeneration. Johnson et al. (182), e.g., observed evidence of neuroinflammation in 28% of their TBI patients at more than 1 year after injury and up to 18 years after insult. In preclinical blast injury studies, microgliosis, usually assessed by alterations in Iba-1 staining, is regularly observed (168, 181, 183–188). Likewise, reactive astrocytes, which may mediate proinflammatory and anti-inflammatory effects, are a common benchmark (181, 189–191). In all of these reports, male laboratory animals were used exclusively. Other evidence of inflammatory signals following TBI is commonly reported. For example, Späni et al. (27) recently summarized their findings from a number of their studies that levels of several cytokines and chemokines were elevated in the brain after closed head injuries, including IL-1β, IL-6, tumor necrosis factor α (TNF-α), IL-10, CXCL1, and CCL2, and sex differences were noted where the concentrations IL-6, TNF-α, and CCL2 levels were higher in female mice after injury, compared with males. Blast exposure likewise results in cytokine responses, which includes IL-1β, IL-6, IL-12, IL-18, IFN-γ, and TNF-α, and chemokines, monocyte chemoattractant protein-1, GRO, and RANTES [e.g., (185, 189, 192–195)]. However, no studies to date that evaluated protein or mRNA changes have examined sex differences in expression in animal models.

## Preclinical Modeling of Blast for the Study of Sex Differences

### The Challenge of Modeling Blast Events

As just reviewed, interpretation of the patient literature on blast effects is a challenge and can at best be viewed as “unsettled” regarding bioeffects and potential differences based on sex. Likewise, there is a dearth of preclinical reports that have investigated SABV. However, for preclinical research of sex differences, this can be viewed as a unique opportunity to get things right. Likewise, there are compelling reasons for getting things right for investigators to recognize the relevance of matching, as best as possible, in-laboratory blast experiments to real-world scenarios. Experimental approaches to preclinical modeling of blast effects then relate not only to present efforts and mandates to evaluate sex differences (26), but also for recognition of potentially important bioeffects from blast. A clear understanding of blast exposure effects has extraordinary relevance to how to direct efforts to treatment, requiring rigorously established models. This section reviews important parameters that have been recognized for their role in reaching valid conclusions for preclinical research studies. Previous approaches, which have not to date so much addressed blast research, have shown how complex sex difference studies can be, and—consequently—strong experimental design is critical to what may be small but significant experimental effects. The majority of publications related to neurobiological effects have employed laboratory shock tubes using compressed gases (40, 196). This will be the emphasis in the discussion below.

Modeling blast effects is a task. Over five decades ago, White (197) summarized the state of the science for understanding “shock and blast biology.” He recognized the vast challenges of outlining the relevant physical and biological parameters for delineating the hazards to man. White emphasized the need for closer collaboration especially between physicists and biologists—although he makes note of critical additional collaboration with engineers, architects, and physicians—for each expert to bring their discipline to bear on this problem. The need for integration continues to be echoed by experts, where only through collaborative efforts between blast physicists and biologists (198), military-relevant and academic researchers (199), and “surgical engineers” (200) and that there can be progress by learning from clinical cases that elucidate what symptoms require mimicking in animal studies (199). The blast neuroscience or neuroendocrinology investigator, then, should seek collaboration and ongoing consultation with the appropriate experts who can immensely improve the quality of the research effort.

### Blast Events

In an explosion, the rapid expansion of detonation products drives a supersonic shock wave into the surrounding air. The ambient air is compressed in microseconds as the shock front passes a location, after which the pressure falls rapidly to pressures below ambient levels over the timescale of milliseconds. The shock front is also associated with an immediate jump in air-flow velocity, or “blast wind,” which can be of hurricane strength, although this also decays rapidly along with the overpressure. The majority of TBIs sustained by blast are classified as mTBI, defined by the Department of Defense as a loss of consciousness <30 min, posttraumatic amnesia for 24 h or less, and alteration of consciousness for a duration <24 h (201). Brain injury resulting from explosive blast occurs as a result of several mechanisms: (1) primary—direct impact on bodily tissues caused by the abrupt variation in air pressure resulting from the blast overpressure wave, (2) secondary—penetrating or blunt injuries as a result of debris set in motion from the blast, (3) tertiary—coup/countercoup injuries resulting from acceleration and deceleration of the body and head or the head/body striking the ground or other object (202–204). Although most blast-induced brain injuries result from primary through tertiary mechanisms, also spoken of are quaternary injuries that result from intense heat (burns) and quinary injuries such as infections, radiation illness, tetanus, and poisoning that are varied and are the result of other injurious factors that are released at the time of the explosion (199).

**Figure 1:**
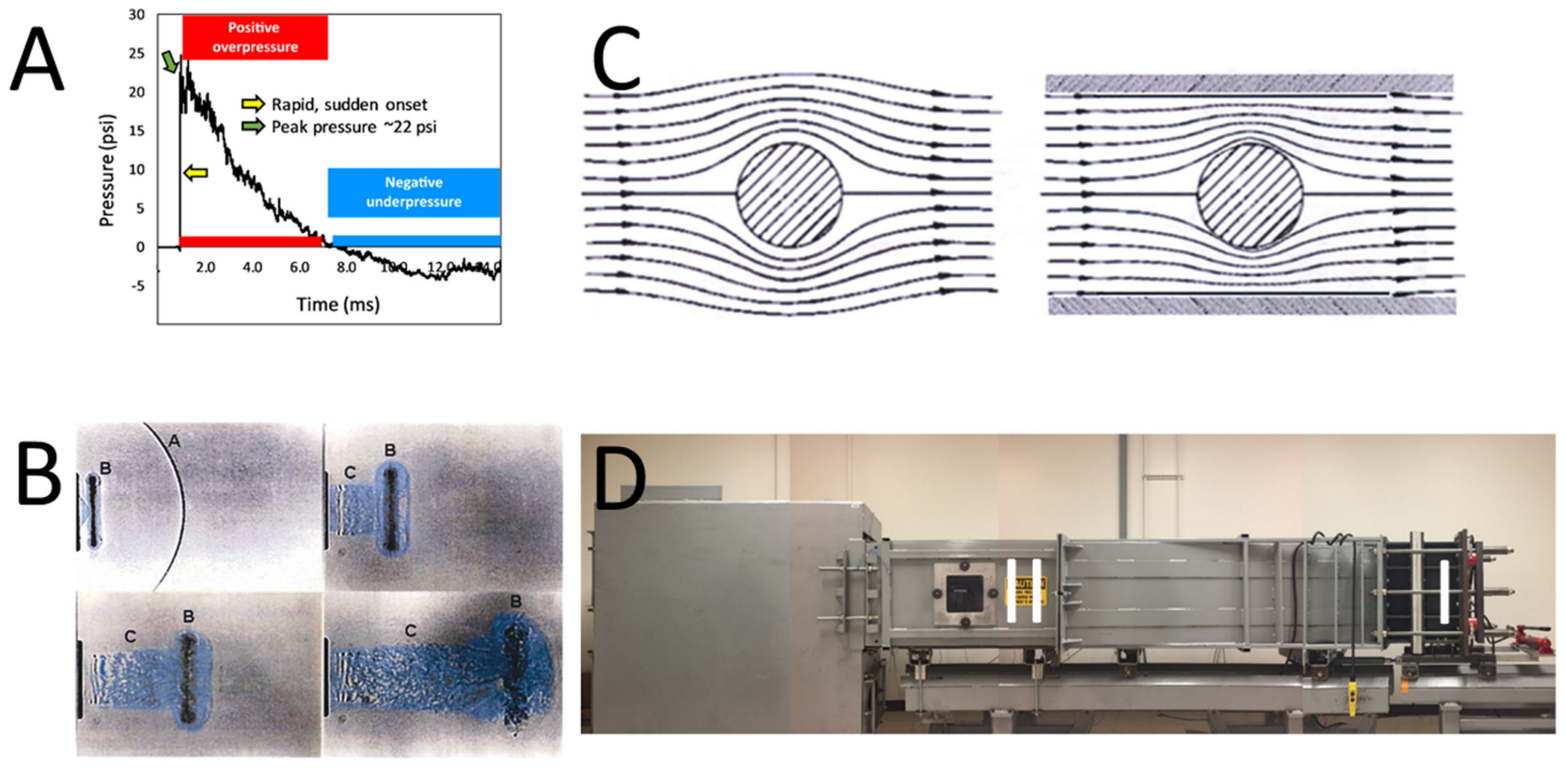
Illustrations for implementation of shock tubes. **(A)** An example of a Friedlander-type shock wave initiated in an ABS. The tube has been previously described (205), and in this setting, a Valmex (7270, Low & Bonar, Martinsville, VA) membrane was employed to generate a shock wave of ~22 psi “peak pressure” (green arrow). The time-pressure trace shows the almost instantaneous change (yellow arrow) in ambient pressure from the shock wavefront, as well as the positive phase (red horizontal bar) and negative phase (blue horizontal bar) that follows as “blast wind.” **(B)** An example of the complex end-jet waveform. The shock wave [A in upper left of photo in **(B)**] emerges from the end of the shock tube and quickly diffracts into a curved front. Following [B in **(B)**] is a “ring vortex” and [C in **(B)**] a venting jet which has a different waveform than the static pressure phase of a Friedlander waveform [from (198)]. **(C)** Illustration of the flow field of a shock wave as it diffracts around a test object. Larger test objects in a confined shock tube can alter the flow of the shock wave, distorting free-field conditions [From (198). **(D)** Photograph of the ABS at the Uniformed Services University of the Health Sciences [cf., (205)]. The illustration depicts several aspects of optimal design of a shock tube. The driver section (I) is distal to the position of the test section (II), where, e.g., an animal would be secured for study. Likewise, the position of the animal is distal to the end of the tube, obviating end-jet effects (including reflected waves) resulting from the emergence of the shock wave from the tube.

In a free-field setting, the Friedlander curve (Figure 1A) is used as the model for an ideal blast wave, and with specific design, this waveform can be replicated by especially designed laboratory shock tubes (206, 207). The key feature of the blast wave is the shock front, causing a nearly instantaneous change in the gas-dynamic properties of the air such as the static pressure, flow velocity, density, and temperature. The shock front thickness is less than a micron translating to a rise time of the order of a nanosecond; this shock front itself is capable of tissue disruption due to the extreme rate of loading. While the human body can endure extremes of pressure (300 psi in the case of “free-divers”), tissue is highly sensitive to rate of change of pressure, in this case in the form of a supersonic wavefront. Following the shock front, the gas-dynamic conditions decay uniformly to below ambient levels (the negative phase) before gradually returning to ambient. The duration of the positive phase is dependent on the scale of the blast being several milliseconds in the case of a typical roadside IED. Simplistically, the static overpressure of the wave causes crushing action, whereas the combination of high-flow velocity and high air density represents the “blast wind” effect causing displacement action and the tertiary blast injury effects described earlier.

Blast physics experts have emphasized the extreme complexity of real-scenario explosions and that while it is one means for setting experimental conditions, including well-designed shock tube studies, the Friedlander wave has been adapted as a model for free-field explosions, but the waveform in no way mimics the high variation and complexity of conditions (208). In real-world scenarios, the target and surrounding objects have a great influence on the blast waveform, and shock wave interactions with surroundings lead to complex reflected waves that can amplify intensity and be followed by secondary shocks and variable negative shock wave phases (208). An explosion above ground, e.g., will cause a complex shock wave due to the effect of the ground reflection. A compound wave structure develops involving a Mach stem with shock wave properties of much greater severity than the incident blast (209). Likewise, when a shock wave encounters a wall or traverses an enclosed space, the reflected wave can be 2–14 times the magnitude of the incident wave (209, 210). For an IED, the shock wave characteristics are altered by a number of interacting factors such as charge shape (e.g., IEDs designed for penetration of vehicles), the encasement of the charge, and the subsurface location that adds tertiary effects from the high-velocity ejecta (dirt, casement, additional components of the IED such as metal shard, toxic and exothermic chemicals). All of these components add immeasurably to the complexity of the injuries; laboratory conditions are simplifications. Nevertheless, the idealized Friedlander-type waveform remains an important reference standard for “free-field” blast exposures for the purposes of laboratory research studies. Although a conventional shock tube was never intended to generate the specially tailored waveform distinctive of explosive blast, within certain important constraints a good approximation can be achieved.

Factors associated with biomechanical differences related to scaling, sex, and age are also of relevance. For example, blast effect sex differences for human males and females have received little attention, but—while there is significant overlap—there are reported average differences in size and skull thickness that can have different consequences on skull flexure during shock wave loading (211–213). Likewise, the skull shape differences are significantly dissimilar for different species used in preclinical study (214), for the determination of sex differences in primates, but mouse differences appear to be trivial (215). Lastly, the focus in blast research has been on younger women and men. The impact of hormone status in older adults and laboratory animals, chronic disease conditions, and aging, as predominant and overriding contributors to morbidity, has not been investigated in blast studies.

### Modeling Preclinical Blast for SABV

For several years now outstanding—and edifying—publications outline details for proper design of shock wave studies (198, 206–208, 216–219). Several of the noted publications, e.g., emphasize the important issue of specimen placement. Most experts indicated that placement of specimens just outside a shock tube is problematic, because either the nature of the shock wave in this position is extremely difficult to characterize, or the shock wave has components that diverge significantly from the conventional Friedlander profile. Specimen position near the exterior of a shock tube results in exposures that are significantly different from a free-field waveform, where the “exit jet” exhibits anomalies (Figure 1B), including multiple peaks, rarefaction waves, and unclear combinations of sonic blast and subsonic effects, including large gradients in flow (198, 220). A second important consideration is the size of the specimen relative to the dimensions of the tube. Referred to as the “presented area,” the specimen should not occupy more than 5–10% of the cross-sectional area of the tube to not impede the steady flow of the shock wave (Figure 1C), where blockage alters the free-field flow of the shock wave and can cause ancillary tertiary effects from specimen acceleration (198, 219). Optimal design of the shock tube can provide a location inside the apparatus (Figure 1D) that minimizes end-tube perturbations, including a strong reflection wave generated when the tube has an open end, and can further control the waveform by “tailoring” to reduce transverse and longitudinal waves inherent in tubes (208). Likewise, when attempting to mimic primary shock wave conditions, securing the specimen is an important additional factor. The restraint system may contribute to test injuries resulting from blast wind effects that result in impacts with the holder (198). Sawyer et al. (221), e.g., emphasize how dynamic pressure can cause head movements, leading to increased staining for glial fibrillary acidic protein, which is a common “confirmatory” injury observation in shock tube publications. In their model, elevations in staining were observed when the head was not restrained, while head fixation, limiting effects to primary shock wave effects, showed no change (222). In addition to the constraints described previously, efforts should be directed to not solely apply a strong air blast as a model for research without ensuring it meets characteristics that are relevant to actual conditions.

### Research Guidelines and Standards for Results Reporting

Related to the need for proper design of shock studies, there has been a recent convergence of views regarding a critical aspect of blast research progress with the paramount need for standardization of results reporting for blast studies (23, 206, 208, 210, 216, 223–227). Clear description of static, dynamic, stagnation, and reflected pressure and how these properties are measured and interpreted are critical. The necessity of standardization of reporting was especially emphasized by experts with concerns regarding the proliferation of blast devices with questionable relevancy to real-life scenarios and the potential for misleading interpretations of biological effects that do not reflect the actualities of blast biology and the difficulties for synthesis and summarizing findings from such laboratories with uncommon shock devices (23, 198, 199, 216, 217, 225).

Indubitably, this challenge of comparability from TBI research endeavors is not restricted to preclinical models of blast. Clinical TBI investigators have been formalizing data reporting since 2008 (228–230), with the formation of the Interagency Common Data Elements Project for TBI, with an updated version described in Hicks et al. (231). A website for clinical study registration and data storage, as a repository permitting eventual secondary meta-analyses by the TBI community, was established by the Federal Interagency Traumatic Brain Injury Research Informatics System for TBI Research (FITBIR; https://fitbir.nih.gov/). The initiative got underway from a Workshop for the Classification of TBI for Targeted Therapies held in October 2007, by the National Institute of Neurological Disorders and Stroke, with the participation of representatives from other groups, including the Defense and Veterans Brain Injury Center and the National Institute on Disability and Rehabilitation Research. The working group initially focused on the limitations of diagnostic criteria and that a pathoanatomical classification system could be the springboard for addressing the heterogeneity of TBIs and for improved systemization for clinical studies and trials (232). A commentary by Dr. John Povlishock, editor of the *Journal of Neurotrauma*, emphasized the importance of this enterprise to basic scientists for their assessment of pathobiology in preclinical research (233). In 2012, FITBIR initiated the effort for a data recording system that employs common data element terminologies.

A subsequent meeting, the Traumatic Brain Injury Preclinical Working Group, was convened to develop a dictionary of common data elements for preclinical studies (234). This group emphasized the importance of the initiative for further “enhancing rigor, reproducibility, and transparency in study performance” in preclinical studies. The CDEs are available as a Preclinical TBI CDE Zip File in Excel format at https://fitbir.nih.gov/content/preclinical-common-data-elements. The Excel files list 61 “Core, Module 1” descriptors (species, animal age, vendor, treatment conditions and outcome measures, etc.) and 41 elements in “Module 6,” specific to blast/shock studies (Table 2). The recent publication of Rodriguez et al. (235) is an excellent example following this scheme. Finally, there are a number of efforts to encourage open data sharing (236), including unpublished data, dubbed “dark data” (237), and efforts to promote preregistration of studies for peer-centered review of studies (238). For good progress in determination of sex differences and blast effects, these initiatives may move the field forward.

**Table 2:** Common data elements for preclinical blast research^*^.

**Title**	**Description**
Blast-induced delivery device	Device used to induce blast injury
Pressure wave type	Friedlander wave is an instantaneous rise in pressure immediately followed by a decay curve; idealized blast in open space; can be reproduced in tube
Detonation type	Material for open field explosions, blast tube explosions
Detonation material quantity	Quantity of material used for open field explosions, blast tube explosions
Driver gas	Gas used to generate overpressure in shock tube
Pressure wave medium	Medium through which blast wave travels to reach target
Distance from detonation	For open-field exposures
Blast tube or column area	Area of the distal end of the blast tube/column or shock tube/column
Blast tube length	Length of the blast tube; use when no membrane is used
Shock tube driven section length	Length of the shock tube driven section; use when membrane present
Membrane/diaphragm thickness	Thickness of membrane between driver and driven sections of shock tube
Membrane/diaphragm burst method	Indicate whether membrane is punctured or allowed to rupture by gas pressure buildup in driver section of shock tube
Membrane/diaphragm burst pressure (shock tube)	Pressure at which the membrane/diaphragm within the shock tube bursts
Tube end configuration	Is the tube end “open” or “closed”
Placement of animal relative to shock tube	Inside or outside the shock tube
Distance between the animal and the tube end	Indicate how far animal is from the end of the shock or blast tube
Animal orientation to the blast wave	Describe positioning of the animal relative to the blast wavefront
Overpressure peak (blast or shock)	Incident pressure
Overpressure rise time	A measure of how rapidly pressure changes from the ambient level to the maximum positive value, defined as the time required for pressure to increase from 10% to 90% of the maximum positive value
Overpressure wave duration (pulse width)	Full width at half maximum amplitude
Impulse	Integration of overpressure with respect to time
Reflective wave overpressure	Pressure measured following reflection or dampening; overpressure following interference
Blast wind pressure	The post-shock or blast wind is important in describing the complete blast wave
Pressure sensor orientation	Location of pressure gauge needed to assess temporal, spatial characteristics of measured pressure
Pressure sensor type	Indicate type of pressure sensor used to characterize, calibrate, and/or record pressure
Pressure sensor sampling frequency	Pressure sensor sampling frequency
Incident pressure time history (image)	Incident pressure time history (image)
Body exposure	Designates whether whole body is exposed to pressure or is partially shielded
Protective shielding	Location
Protective shielding type	Nature of material used for shielding
Reflective surfaces (where and type)	Indicates the presence and nature of reflective or dampening surfaces integrated into blast wave path
Primary blast effects	Methodology employed to isolate primary blast effects from secondary, tertiary, or quaternary effects
Secondary blast effects type	Secondary blast effects include the effects of any projectile, including fragments of debris, propelled by the blast that penetrates the skin. This may be modeled with a blast (primary blast effect) or in isolation to mimic the secondary blast effects associated with a blast. Cross reference with penetrating models of brain injury as appropriate
Secondary blast effects specifications	Entered to further explain “secondary blast effect type.”
Tertiary blast effects type	Tertiary blast effects describe when explosion propels body and brain is injured due to acceleration and/or impacts the ground or a surrounding object. For animal models, could be used to describe the head hitting the ground or object, or ground or object hitting head. For small objects, use secondary blast effects
Tertiary blast effects specifications	Provide further explanation of methods used to induce tertiary injury and/or methodology to measure resultant forces or accelerations. Cross reference with blunt force and/or acceleration model CDEs as necessary. For head impact only (i.e., no blast), use appropriate CDE (e.g., weight drop model)
Quaternary blast effects	Quaternary blast effects include toxic gas inhalation, thermal exposure, flash burns, microwave heating, electromagnetic fields
Systemic injury	Measures of systemic inflammation/stress as a result of the blast (including primary, secondary, tertiary, quaternary effects)
Extracranial injuries	Injuries other than brain injury that occurs as a result of the blast (including primary, secondary, tertiary, quaternary effects)
Blast-induced specific preinjury surgical procedures	Description of any presurgical procedures specific to the blast-induced neurotrauma model
Blast-induced specific postinjury surgical procedures	Description of any postsurgical procedures specific to the blast-induced neurotrauma model

* *From https://fitbir.nih.gov/content/preclinical-common-data-elements*.

Of added high relevance for preclinical blast research is the framework of the NATO Task Group, HFM-234 (220). This document resulted in the dissemination of useful guidelines, including rules for more detailed description of the blast (or shock) exposure device (219) and the specifics of animal modeling (239). In addition to allowing comparisons across laboratories, improved standardization and description of conditions can lead to improvement of data quality where the guidelines permit funding bodies to better evaluate the experimental plan and design of proposals, and journal reviewers and editors to have a better sense of the quality of reported findings (239).

The original publications should be consulted for full discussion, but some of the most salient challenges are outlined here. Table 3 is a summary checklist for experimental planning; many of the queries in the checklist overlap with the Common Data Elements in Table 2, but it is included here because there is additional emphasis on investigator review of study rationale and description of the shock/blast-generating apparatus. Investigators of blast effects on preclinical models of sex differences should first consider the details for inducing blast overpressure [cf., (219)].

**Table 3:** Checklist for experimental planning of preclinical blast studies^*^.

1) Start with a clearly stated question you wanted to answer 2) What was the rationale for selecting the model you did? 3) The model must be a valid model for the question 4) What parameters will be measured (both biomechanical and biological) and how are they related to real-life conditions or other published work? 5) Can you vary the parameters accurately within field-relevant range, so you can examine the range of observed injuries? 6) Have recognition that there are limits to your model so that results are not overinterpreted 7) Need to ask if these changes you see in the animal model are changes we would see in humans 8) Rationale for using the animal model, the species, weight, gender, age, etc., a description of all the things that matter, i.e., 20- vs. 60-kg pig is important as well as how firmly they are fixed 9) Expected kinetic therefore the rationale for choosing specific time points. Justification of your end points. This may be species specific? 10) Where are the animals placed in a test field? Show clearly in a diagram with respect to loading source. Rationale for this. In the guide will describe drawbacks or issues with placing an animal in certain areas of the tube 11) Have to give the relevant exposure for the question they are answering, not overexposing or underexposing the animals for the problem they are trying to answer 12) Can you relate observed pathophysiological changes as a function of external loading and different time points? 13) Justification for the use of a certain technique, e.g., use of explosives instead of compressed gas for primary blast experiments 14) Justify the specific placement and binding of the animal in the experimental model through direct pressure, acceleration, and strain measurements on the animal or animal surrogates 15) A plan for the statistics, and where possible a power calculation, and estimation of n numbers 16) Can rodents be used or would gyrencephalic species, such as ferrets or pigs, be needed? 17) Will the skull thickness, head shape, and orientation of the animals affect the result when translated to an erect human with face forward to blast?

* *Table 1 in Appendix J1 from NATO Health Factors and Medicine (HFM) Research Task Group (RTG) HFM-234 (220)*.

The application of blast, whether using free-field exposure or a laboratory-based apparatus that employs blast (explosion) or compressed gas as the driver, must be recognized for its complexity of model application, the inherent pitfalls in each model, and the onus for understanding and communicating exposure metrics. A detailed description of the design of the experimental setting should be documented, including the dimensions of the free-field conditions or the shock/blast tube, for investigators to have a clear sense of the exposure conditions. A published description of the environment becomes a permanent record of intervening effects permitting meta-analyses through standard reporting. Full reportage also allows documentation of existing or potential experimental artifacts, such as reflective and blockage effects from the surroundings (including gauge or animal holder interference) and potential constraining factors such as shock tube dimensions, the size, location, orientation, fixation/restraint conditions of the study specimen(s), and exposure conditions of the test specimen(s) in relation to the overpressure source (209, 217, 219). The guidelines address additional considerations. Is the stimulus reproducible and controllable, and how have the conditions been quantified? What were the conditions pertaining to reflection? What is the intended nature of the injury? If the focus is primary blast, what conditions are in place to mitigate secondary and tertiary effects (207, 239)? Finally, other considerations must be heeded, including choice of recording devices that accurately allow spatial assessments of static pressure for above-ambient pressure (207) and total pressure from the motion of gas (dynamic pressure) with the static pressure, assessed by Pitot tube (219). Do measurement devices have adequate sensor bandwidth to accurately record changes (219, 240)? The aforementioned publications offer excellent overviews for experimental design and emphasize the point of the studies—translation of results—which demands validation of findings and their relevance to real-event scenarios and accurate and correct identification of underlying pathology as an entrée to therapeutic translation.

For the role of sex in underlying biological responses, the field of laboratory blast studies *is ripe to get things right*. As noted by the Traumatic Brain Injury Preclinical Working Group, preclinical blast research studies are presently an “immature research area” where the lack of more substantial clinical information as a guide to basic hypothesis-driven research is a challenge; the causal effects following blast are emerging but still uncertain, and there are no commonly accepted injury devices (234), as is seen in other models employed in SABV with hundreds of publications. Challenges in the future related to blast and SABV will demand clarification of potential “differences in metabolism, pharmacokinetics, receptor distribution and activity, enzyme activity, and [how] ongoing hormonal interactions may affect whether a particular intervention exhibits important neurological properties” (241). Rigor and standardization are critical for furthering our understanding of sex differences, which are complex and potentially of smaller effect sizes, but critical for translating findings for clinical relevance.

## Author Contributions

All authors listed have made a substantial, direct and intellectual contribution to the work, and approved it for publication.

## Conflict of Interest

The authors declare that the research was conducted in the absence of any commercial or financial relationships that could be construed as a potential conflict of interest.
